# Postoperative sarcopenia in older patients with hip fractures: incidence and associated risk factors in a retrospective cohort study

**DOI:** 10.3389/fmed.2026.1766120

**Published:** 2026-04-22

**Authors:** Hang Yin, Chao Qin, Qi Tang, Guoli Wu, He Diao

**Affiliations:** 1 Baicheng Medical College, Baicheng, Jilin, China; 2 Tongliao People's Hospital, Tongliao, Inner Mongolia, China; 3 The Fifth Affiliated Hospital of Harbin Medical University, Daqing, China

**Keywords:** epidemiology, geriatric population, hip fracture, postoperative sarcopenia, risk factors

## Abstract

**Objective:**

Sarcopenia significantly impacts the quality of life in older adults. Given the lack of large-scale studies on risk factors for postoperative sarcopenia in older hip fracture patients, this study investigated its incidence and associated risk factors.

**Materials and methods:**

This retrospective study enrolled older patients who were admitted for surgical treatment of hip fractures.

**Results:**

Out of the 325 enrolled patients, 264 (81.23%) were identified as being at risk for postoperative sarcopenia. The average sarcopenia score was 5.01 ± 1.61. Significant differences (*p* < 0.05) were observed across age, marital status, education levels, living arrangements, exercise habits, masticatory function, the number of chronic diseases, albumin levels, and activities of daily living (ADL) scores. Multivariate regression analysis identified age, education levels, activities of daily living (ADL) scores, and masticatory function as independent risk factors for sarcopenia.

**Conclusion:**

This study identified several risk factors for sarcopenia following hip fracture surgery in older adults, providing a basis for targeted clinical interventions to mitigate postoperative muscle loss.

## Introduction

Aging in older adults results not only in bone loss but also in declines in muscle strength and mass. Early signs of sarcopenia, an age-related muscle-wasting syndrome, are often subtle and easily overlooked in this population (1). Sarcopenia commonly complicates recovery after hip fracture surgery in older adults, affecting over 45% of patients (2). As the global population ages rapidly, the number of hip fracture surgeries continues to rise. Consequently, the incidence of post-surgical sarcopenia has steadily increased worldwide over the past decade, raising significant concerns within healthcare systems and social security frameworks (3). Epidemiological studies indicate that post-surgical sarcopenia substantially increases the 30-day mortality rate, ranging from 9 to 23.7% (4–7). It also prolongs hospital stays by 56% and significantly heightens the risk of hospital readmissions (8). Factors exacerbating this issue include advanced age, multiple comorbidities, significantly reduced organ functional reserve, the trauma of the fracture and surgery, and prolonged limb immobilization after injury. The most cost-effective strategy to mitigate these adverse outcomes involves identifying common, potentially modifiable risk factors, particularly those amenable to change. This approach allows for early, targeted interventions to reduce the occurrence of post-surgical sarcopenia.

Over the past decade, numerous studies have examined potential risk factors for postoperative sarcopenia in older adults after hip fracture surgery (9–11); these factors include sex, age, body mass index, chronic obstructive pulmonary disease, hypoalbuminemia, anemia, and the number of comorbidities (12–16). However, significant gaps remain in the research. The majority of studies focus on Western populations and isolated clinical indicators, often overlooking comprehensive functional assessments. In China, surgical treatment for hip fractures is often delayed by several days, which deviates from the recommended protocols for early intervention (17, 18). These delays may change the epidemiology of postoperative sarcopenia. Large-scale, comprehensive studies investigating the risk factors for postoperative sarcopenia in Chinese older adults with hip fractures remain critically scarce. Addressing this gap is essential to optimize clinical pathways.

**Figure 1:**
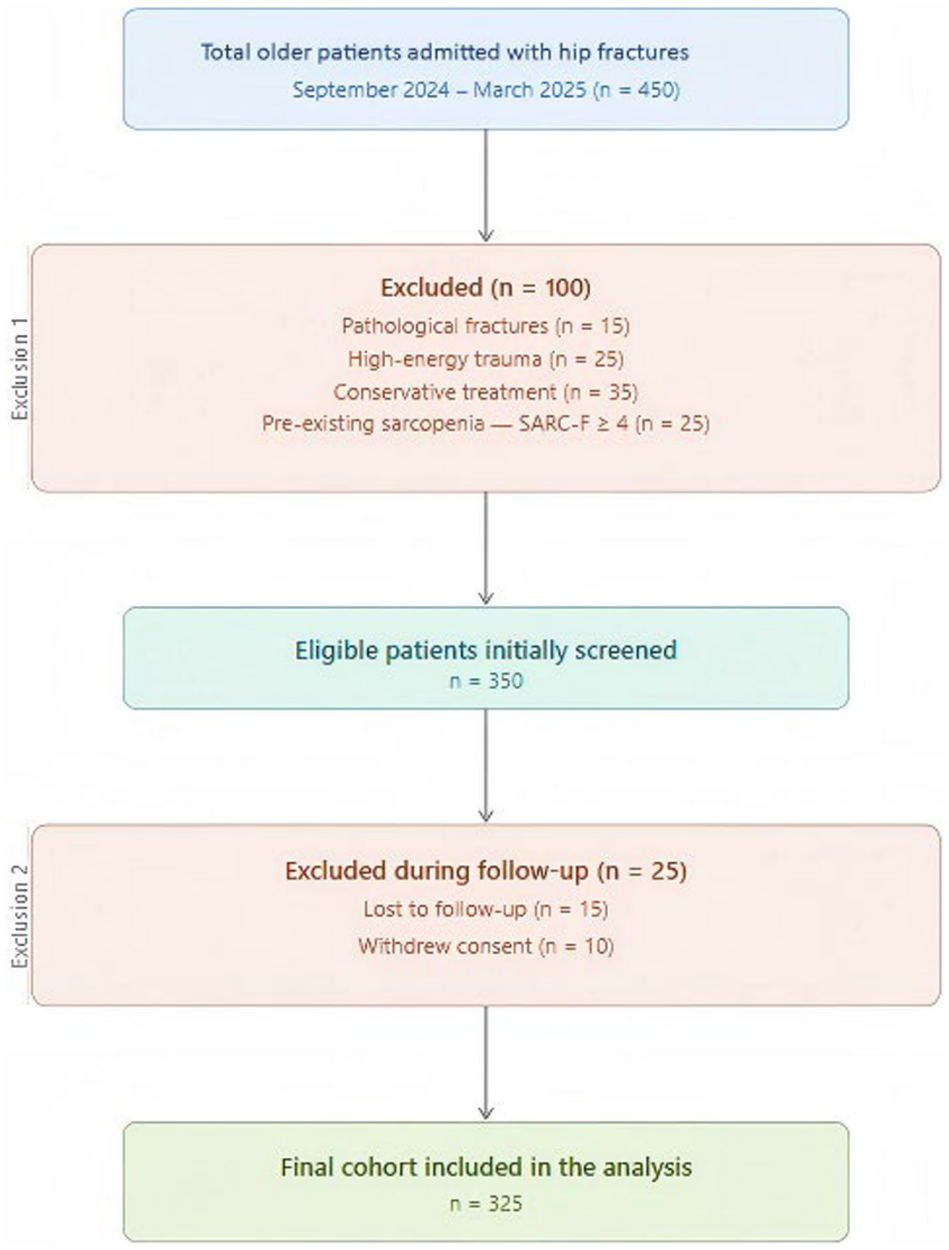
Flowchart of the screening process for the study participants.

This study aims to (1) determine the incidence of postoperative sarcopenia during hospitalization and (2) identify risk factors for sarcopenia in older patients undergoing hip fracture surgery by evaluating sociodemographic characteristics and comprehensive functional profiles. Ultimately, the results will support an evidence-based framework for targeted clinical interventions (Figure 1).

## Materials and methods

This retrospective study was conducted from September 2024 to March 2025. Participants included patients aged 60 years or older who sustained an acute hip fracture from low-energy trauma and were scheduled for either joint replacement or internal fixation surgery. Exclusion criteria were fractures resulting from moderate- or high-energy trauma, non-acute fractures, pathological fractures, multiple fractures or polytrauma, patients treated conservatively, revision surgeries, any reoperations, and long-term use of immunosuppressants. As hip fractures are acute, prospective baseline assessments were not feasible. To identify preexisting sarcopenia, a retrospective baseline evaluation was performed at admission. Patients or their primary caregivers completed the SARC-F questionnaire, reflecting the patient’s physical and functional status 1 month before the fracture. Individuals with a score ≥ 4 were excluded. The incidence of postoperative sarcopenia was then assessed using three-month post-discharge follow-up records. A total of two specially trained orthopedic nurses conducted these assessments either by telephone or during routine outpatient visits. All data were double-entered and cross-verified to minimize errors, with discrepancies resolved through discussion to reach a consensus. The study received approval from the School of Nursing at Baicheng Medical College and adhered strictly to relevant regulations and the principles of the Declaration of Helsinki. Informed consent was obtained from all participants.

### Definition of sarcopenia

In 2009, the European Working Group on Sarcopenia in Older People (EWGSOP) defined sarcopenia and established its diagnostic criteria. They identified sarcopenia as a syndrome characterized by the progressive loss of skeletal muscle mass and decreased muscle strength. The diagnostic criteria include three main aspects: Muscle strength, muscle mass, and physical performance (19). This study employed the SARC-F questionnaire, developed by Malmstrom et al. in 2013 (20), to evaluate the risk of sarcopenia. This tool assesses five components through self-report: Muscle strength, walking ability, stair climbing, history of falls, and chair rising ability. Each component is scored from 0 to 2 points, with a maximum total score of 10 points. A total SARC-F score of ≥ 4 points indicates probable sarcopenia, with higher scores suggesting greater severity.

### Variables of interest

The variables of interest in this study included age, sex, ethnicity, marital status, educational level, living arrangement, medical payment method, exercise habits, albumin and hemoglobin levels, masticatory function, types of chronic diseases, activities of daily living (ADL) score, and nutritional status.

### Statistical analysis

Data in this study were double-entered and organized using SPSS 26.0 and Excel, followed by statistical analysis of the questionnaire data. A *p*-value of < 0.05 was considered statistically significant. The incidence of postoperative sarcopenia was calculated using the 3-month post-discharge follow-up data of the participants. Means and standard deviations were used to describe numerical or quantitative data, while frequencies and constituent ratios were used to describe categorical or count data. Since the measured data followed a normal distribution, one-way analysis of variance (ANOVA) or *t*-tests were used. Variables that were statistically significant in the univariate analysis were entered into a multiple linear regression model to identify independent risk factors for postoperative sarcopenia. Before modeling, potential multicollinearity among variables was assessed using the variance inflation factor (VIF). As the proportion of missing clinical data was extremely low, patients with incomplete follow-up records were excluded from the final analysis.

## Results

During the study period, 450 patients were recruited, and initial screening identified 350 eligible candidates. After excluding 25 individuals who were lost to follow-up or withdrew from the study, the final analysis cohort comprised 325 participants. The cohort’s mean age was 72.44 ***±*** 6.59 years (range: 62–90 years). Demographically, 195 participants (60.0%) were female, 206 (63.4%) were married, and 34 (10.5%) lived alone. Baseline laboratory tests showed that 91 participants (28.0%) had normal serum albumin levels (≥37 g/L), while 146 (44.9%) had hemoglobin levels ≥110 g/L. Furthermore, 213 participants (65.5%) presented with one or two comorbidities. Table 1 summarizes the cohort’s baseline sociodemographic and clinical characteristics.

**Table 1:** General sociodemographic characteristics of the study participants (*n* = 325).

Item	Group	Number of cases (*n*)	Proportion (%)
Age	60~69	131	40.4
	70~79	149	45.8
	80~	45	13.8
Sex	Male	130	40.0
	Female	195	60.0
Ethnicity	Han	177	54.5
	Ethnic minorities	148	45.5
Marital status	Married/with spouse	206	63.4
	Unmarried/without spouse	119	36.6
Education level	Junior high school or below	223	68.6
	High school/technical secondary school	55	16.9
	College or above	47	14.5
Living arrangement	Living with spouse and children	169	52.0
	Living with spouse	32	9.8
	Living with children	90	27.7
	Living alone	34	10.5
Monthly income per capita	<2000 CNY	79	24.3
	2000–4,000 CNY	216	66.5
	>4,000 CNY	30	9.2
Previous occupation	Government/Institutional Personnel	46	14.2
	Worker	142	43.7
	Farmer	91	28.0
	Other	46	14.1
Medical payment method	Urban employee/resident medical insurance	218	67.0
	Rural cooperative medical insurance	85	26.2
	Other	22	6.8
Exercise habit	Yes	288	88.6
	No	37	11.4
Assistive devices	None	223	68.6
	Yes	102	31.4
Albumin level	<37 g/L	234	72.0
	≥37 g/L	91	28.0
Hemoglobin level	<110 g/L	179	55.1
	≥110 g/L	146	44.9
Chewing function	Good	139	42.8
	Impaired	186	57.2
Chronic diseases	None	52	16.0
	1 ~ 2 types	213	65.5
	≥3 types	60	18.5
Activities of daily living (ADL) score	Severe dependence	6	1.8
	Moderate dependence	20	6.2
	Mild dependence	256	78.8
	Complete independence	43	13.2
Nutritional status	Malnutrition	137	42.2
	Nutritional risk	161	49.5
	Normal nutritional status	27	8.3

Medical payment terms are translated based on China-specific healthcare system classifications. CNY = Chinese Yuan.

Univariate analysis showed that age, marital status, educational level, living arrangements, physical activity, albumin level, masticatory function, number of comorbidities, and ADL score were significantly associated with postoperative sarcopenia (all *p* < 0.05; Table 2).

**Table 2:** Univariate analysis of factors associated with sarcopenia among the participants (*n* = 325, mean ± SD).

Item	Group	Total	Score (mean ± SD)	*t/F*	*P-value*
Age	60~69	131	4.63 ± 1.40	41.955	<0.001
	70~79	149	4.79 ± 1.45		
	80~	45	6.82 ± 1.50		
Sex	Male	130	4.86 ± 1.71	−1.353	0.177
	Female	195	5.11 ± 1.54		
Ethnicity	Han	177	4.89 ± 1.57	−1.500	0.134
	Ethnic minorities	148	5.16 ± 1.65		
Marital status	Married/with spouse	206	4.77 ± 1.49	−3.417	0.001
	Unmarried/without spouse	119	5.42 ± 1.73		
Education level	Junior high school or below	223	5.36 ± 1.68	19.437	<0.001
	High school/technical secondary school	55	4.42 ± 1.18		
	College or above	47	4.04 ± 0.98		
Living arrangement	Living with spouse and children	169	4.80 ± 1.51	11.023	<0.001
	Living with spouse	32	4.66 ± 1.43		
	Living with children	90	5.00 ± 1.61		
	Living alone	34	6.41 ± 1.58		
Medical payment method	Urban employee/resident medical insurance	218	5.07 ± 1.60	1.671	0.190
	Rural cooperative medical insurance	85	4.76 ± 1.68		
	Other	22	5.36 ± 1.40		
Exercise habit	Yes	288	4.85 ± 1.44	−3.607	<0.001
	No	37	6.22 ± 2.24		
Albumin level	<37 g/L	234	5.14 ± 1.68	2.517	0.013
	≥37 g/L	91	4.68 ± 1.37		
Hemoglobin level	<110 g/L	179	5.12 ± 1.77	1.376	0.170
	≥110 g/L	146	4.88 ± 1.38		
Chewing function	Good	139	4.32 ± 1.29	−7.381	<0.001
	Impaired	186	5.52 ± 1.64		
Chronic diseases	None	52	4.40 ± 1.36	15.342	<0.001
	1 ~ 2 types	213	4.90 ± 1.52		
	≥3 types	60	5.93 ± 1.75		
Activities of daily living (ADL) score	Severe dependence	6	7.67 ± 1.966	17.475	<0.001
	Moderate dependence	20	6.75 ± 1.713		
	Mild dependence	256	4.90 ± 1.476		
	Complete independence	43	4.49 ± 1.470		
Nutritional status	Malnutrition	137	5.18 ± 1.690	1.376	0.254
	Nutritional risk	161	4.88 ± 1.547		
	Normal nutritional status	27	4.89 ± 1.528		

In the multiple linear regression analysis, age, educational level, ADL score, and masticatory function were included in the regression equation for sarcopenia. The regression model was statistically significant (*F* = 18.372, *p* < 0.001). Table 3 summarizes the regression analysis of risk factors for sarcopenia in the study population.

**Table 3:** Regression analysis of risk factors for sarcopenia.

Variables	Unstandardized B	Coefficients std. error	Standardized coefficients beta	t	*P*	*VIF*
(Constant)	4.530	1.514		2.993	0.003	
Age	0.036	0.015	0.147	2.357	0.019	1.860
Marital status	0.342	0.324	0.103	1.057	0.291	4.531
Living arrangement	−0.133	0.146	−0.091	−0.9096	0.364	4.808
Education level	−0.328	0.107	−0.150	−3.071	0.002	1.144
Exercise habit	−0.038	0.270	−0.008	−0.142	0.887	1.371
Albumin level	−0.219	0.172	−0.061	−1.272	0.204	1.110
Chewing function	0.633	0.173	0.195	3.661	<0.001	1.362
Chronic diseases	0.079	0.145	0.029	0.546	0.585	1.342
Activities of daily living (ADL) score	−0.032	0.006	−0.298	−5.015	<0.001	1.696

Age: Raw data entry. Marital Status: Unmarried/Without Spouse = 0, Married/With Spouse = 1. Living Arrangement: Living Alone = 1, Living with Children = 2, Living with Spouse = 3, Living with Spouse and Children = 4. Education Level: Junior High School or Below = 1, High School/Technical Secondary School = 2, College or Above = 3. Exercise Habit: No = 0, Yes = 1. Albumin Level: Raw data entry. Chewing Function: Impaired = 0, Good = 1. Chronic Diseases: None = 1, 1~2 types = 2, ≥ 3 types = 3. Activities of Daily Living (ADL) score: Raw data entry.

## Discussion

Sarcopenia is a serious complication in older patients following hip fracture surgery, often leading to adverse clinical outcomes (21). This large-sample study systematically analyzed comprehensive risk factors to generate reliable and clinically significant results. The data revealed a postoperative sarcopenia incidence of 81.23%, with age, educational attainment, ADL score, and masticatory function identified as key influencing factors.

In this study, 81.23% of the cohort met the criterion for sarcopenia (SARC-F score ≥ 4) at the three-month postoperative assessment. This prevalence exceeds rates reported in comparable studies; two main factors may explain the higher rate. First, the SARC-F questionnaire relies entirely on patients’ subjective evaluations. By 3 months after discharge, many patients develop a fear of falling (FOF), which limits participation in rehabilitation and reduces functional mobility below expected mid-recovery levels. This avoidance can progress to disuse syndrome, thereby inflating self-reported SARC-F scores. Second, surgical interventions for Chinese patients are often delayed relative to Western practice, and such delays can predispose patients to early postoperative muscle atrophy.

Research indicates that advanced age serves as an independent risk factor for sarcopenia, with notable differences in sarcopenia scores across various age groups. Specifically, the severity of muscle loss progressively increases among older adults (22), with the highest scores observed in the cohort aged ≥80 years, a difference that was statistically significant (*p* < 0.01). Key contributing factors include age-related declines in overall health, as well as impaired digestion and absorption, which lead to suboptimal health status and compromised muscle function. Furthermore, advanced age often correlates with restricted mobility, decreased participation in moderate-to-vigorous physical activity, and prolonged periods of sedentary behavior or bed rest, all of which accelerate the decline in muscle mass and strength. International studies support the assertion that advanced age is a significant predictor of sarcopenia in older adults (23). Consequently, it is recommended that older postoperative patients engage in suitable physical activity and enhance social engagement to preserve muscle mass and mitigate decline. In addition, improved screening and identification of sarcopenia in the oldest-old patients are essential for preventing associated adverse outcomes (24).

This study demonstrated a statistically significant inverse correlation between higher levels of educational attainment and lower sarcopenia scores (*p* < 0.01), aligning with the findings reported by Jiang (25). This association may be mediated through several pathways. Individuals with advanced education typically possess superior overall competencies, fostering proactive health-seeking behaviors, including the pursuit of disease-specific knowledge and preventive guidance. Their enhanced ability to utilize digital resources and literature for health information acquisition, coupled with effective communication with healthcare providers, contributes to robust self-management capabilities. Furthermore, higher educational levels are associated with superior analytical comprehension and executive function compared to lower educational levels. This cognitive advantage, as corroborated by Cabett et al. (26) and Tamura et al. (27), represents a significant socioeconomic determinant influencing sarcopenia risk.

Sarcopenia scores differed significantly among patients with varying levels of functional independence (*p* < 0.01), consistent with the findings of Silva et al. (28). Activities of daily living (ADL) reflect an older adult’s ability to perform routine tasks and correlate strongly with lower extremity muscle strength. Previous research has identified reduced lower limb strength as the principal cause of mobility loss in older adults (29). Therefore, sarcopenia management should prioritize patients with impaired mobility. Postoperative rehabilitation protocols should progressively incorporate resistance training and include early psychological interventions to reduce fear of falling (FOF) and slow the progression of sarcopenia.

In the present study, sarcopenia scores varied significantly according to masticatory function (*p* < 0.01). This finding aligns with Suzuki et al. (30), who reported a higher incidence of sarcopenia among individuals with impaired oral function. Consequently, adequate mastication is crucial for maintaining nutritional status and delaying sarcopenia onset in older adults (31). Umeki et al. (32) identified masticatory muscle mass as a potential predictor of sarcopenia, reporting that greater muscle thickness was associated with a lower risk. Together, these results underscore the critical link between masticatory performance and nutritional intake. Preserved mastication enables older adults to consume a varied, balanced diet. For patients with impaired mastication, providing texture-modified, nutrient-dense foods is essential to maintain key nutrients, particularly vitamin D, thereby mitigating sarcopenia risk.

### Limitations

The primary strength of this study lies in its inclusion of a wide range of exploratory risk factors to analyze their association with sarcopenia in older patients following hip fracture surgery, within a substantially large sample cohort. However, there are some limitations. First, the single-center, retrospective design depends on the completeness and accuracy of historical medical records. To reduce data bias, we implemented double data entry and cross-validation protocols. Second, our primary outcome was assessed at 3 months post-discharge. We selected this timeframe to account for two factors: The influence of musculoskeletal comorbidities on physical function and the established timeline for functional recovery in patients aged 65 years or older following a hip fracture. This relatively short follow-up may not have captured the long-term trajectory of sarcopenia. Therefore, future prospective, multicenter studies with longer follow-up are necessary to fully elucidate the long-term trajectory of postoperative sarcopenia. Third, as with other logistic regression analyses, residual confounding remains a concern because many variables were unmeasured or could not be quantified. For example, post-discharge nutritional intake and adherence to home-based rehabilitation were not strictly monitored, and both factors can significantly influence muscle mass recovery. Fourth, this study primarily assessed postoperative sarcopenia in older patients with hip fractures using the SARC-F questionnaire. Although the SARC-F questionnaire is widely used for screening, our study lacked systematic objective measures, such as dual-energy X-ray absorptiometry (DXA) for muscle mass or dynamometry for muscle strength. Consequently, relying solely on patient or caregiver reports of functional limitations may have introduced bias because respondents’ educational backgrounds can influence their responses. In addition, older adults often develop a fear of falling (FOF) after a hip fracture. This psychological barrier markedly impeded postoperative rehabilitation, increased the overall risk of sarcopenia, and may have skewed the subjective SARC-F assessments. Therefore, future prospective studies incorporating objective diagnostic criteria for muscle mass and strength are warranted to validate these findings.

## Conclusion

This study identified an 81.23% incidence of sarcopenia at 3 months postoperatively. Patient age, educational level, ADL score, and masticatory function were identified as significant factors influencing postoperative sarcopenia. These findings enhance understanding of sarcopenia management in older patients after hip fracture surgery and highlight key risk factors associated with its development.

## Data Availability

The original contributions presented in the study are included in the article/supplementary material, further inquiries can be directed to the corresponding author.
